# Effect of Distillation Time on the Yield and Chemical Composition of Leaf Essential Oil from *Abies koreana*

**DOI:** 10.3390/plants15071123

**Published:** 2026-04-07

**Authors:** Chanjoo Park, Nahyun Kim, Soo-Kyeong Jang, Mi-Jin Park

**Affiliations:** Forest Industrial Materials Division, Forest Products and Industry Department, National Institute of Forest Science, Seoul 02455, Republic of Korea; chanjoopark515@korea.kr (C.P.); knh1125@korea.kr (N.K.); skjang05@korea.kr (S.-K.J.)

**Keywords:** distillation time, essential oils, *Abies koreana*, yield, bioactive components

## Abstract

Distillation time (DT) is a key parameter influencing yield and chemical composition, and its optimisation is crucial for production. This study is the first laboratory-scale investigation of the effect of distillation time (DT) on the leaf essential oils of *Abies koreana*, aiming to maximise oil yield and target bioactive components for cosmetic applications. Essential oils were obtained by hydrodistillation at 14 DTs (1, 3, 5, 10, 20, 40, 80, 120, 160, 200, 240, 280, 360, and 480 min), and the yields, chemical profiles, and fragrance characteristics were comparatively analysed. The control (exhaustive hydrodistillation: 20 h) yielded 2.82% and was dominated by *D*-limonene, bornyl acetate, and camphene. The contents of bioactive compounds associated with whitening and anti-wrinkle activities (α-pinene, *D*-limonene, borneol, and bornyl acetate) varied markedly with DT. The highest oil yield was obtained at 80 min (0.30 ± 0.01%), while the targeted components were achieved at 80–160 min. Heatmap-based multivariate analysis revealed distinct compositional differences between oils distilled at 80 min and 160 min, with DT shifting fragrance profiles from fresh, monoterpene-rich notes (linalyl acetate, camphor, and fenchol) to longer-lasting, sesquiterpene-dominated aromas (α-bisabolol and β-eudesmol). Therefore, distillation time significantly influenced *A. koreana* oil, with shorter distillation (80 min) maximising yield and longer distillation (80–160 min) enriching bioactive components for cosmetic applications.

## 1. Introduction

*Abies koreana* Wilson (Korean fir) is a fir native to the higher mountains of South Korea, including Jeju Island. It grows at altitudes of 1000–1900 m in temperate rainforests with high rainfall and cool, humid summers, as well as heavy winter snowfall [1]. This species has traditionally been used in folk medicine for the treatment of colds, stomachaches, indigestion, rheumatic diseases, and cardiovascular and pulmonary disorders [2]. Furthermore, needle oils are used industrially by soap and perfume manufacturers to add pleasant odours to their products [3]. *A. koreana* oils are extracted from various parts such as the leaves, cones, twigs, and seeds. Specifically, Korean fir seed oils obtained by hydrodistillation (3.8–8.5% yield) are dominated by monoterpenes (70–95%) and oxygenated monoterpenes (1–20%) [4]. Hydrodistilled *A. koreana* leaf oil (0.9% *v*/*w*) is characterised by high levels of bornyl acetate (30.35%) and limonene (18.95%) [5]. In contrast, cone oil is mainly composed of α-pinene (36.1%), limonene (26.5%), and bornyl acetate (10.9%), whereas twig oil is dominated by bornyl acetate (27.2%), limonene (19.4%), and camphene (17.0%) [6]. The biological activities of *A. koreana* oils were reported to include antimicrobial [7], anti-inflammatory [5], anti-wrinkle and whitening effects [8]. Zhang et al. [9] reported that the amount of bioactive ingredients in natural products such as essential oils is generally small, and the extraction and isolation of the active ingredients can be a time-consuming process, which hinders the industrial application of essential oils in commercial use. Therefore, there is an urgent need to optimise distillation conditions to effectively target bioactive compounds in *A. koreana* oils as functional cosmetic ingredients.

The oil yield and chemical profile of aromatic plants can be affected by various factors, including but not limited to environmental conditions, agronomic factors, and distillation techniques [10]. There are several studies of the effect of DT on commercial essential oils, such as those of oregano [11], lavender [12], and fennel [13]. Essential oils are complex mixtures, and their constituents are extracted at different times during distillation depending on molecular weight, boiling point, and volatility. In particular, nearly 5% of the dry matter is made up of essential oils, which are important secondary metabolites possessing the biological activities together with a unique fragrance [14]. It is important to maximise the oil yield for a profitable essential oil industry. Understanding the effects of DT on essential oil yield and the chemical profile might allow producers of these commercial essential oils to increase production and engineer chemical compositions of the oils while decreasing the energy required for distillation [15]. There is no previous comprehensive study on the effect of DT on *A. koreana* oils. Furthermore, investigations into the influence of distillation parameters on essential oils remain relatively limited in coniferous species, including members of the genus *Abies*, despite the increasing interest in them as sources of bioactive essential oils. Establishing distillation conditions for *A. koreana* oils that target specific biological activities would provide fundamental data for the transition toward commercial production.

This study evaluated the effects of DT on *A. koreana* oils in terms of oil yield, chemical composition, and fragrance characteristics to identify suitable distillation conditions for their application as functional cosmetic ingredients. Hence, the determination of the suitable DT provides a practical strategy for controlling both oil yield and quality, thereby enhancing the feasibility of the commercial production of *A. koreana* oils.

## 2. Results

### 2.1. The Yield and Chemical Profile of A. koreana Oils (Control) Extracted by Hydrodistillation

**Figure 1:**
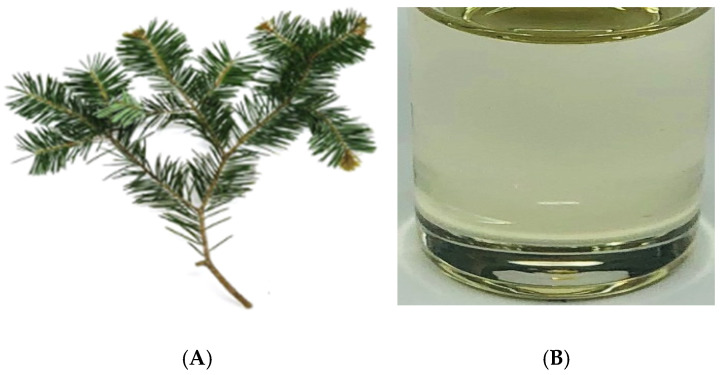
Fresh leaves (**A**) and the colour of leaf oils (**B**) of *Abies koreana*.

The essential oil of *A. koreana* used as the control in this study was extracted until no further oil could be obtained. No additional essential oil was detected after a 20 h of DT; therefore, the process was terminated at that time. The oil yield of the control was 2.82%. The *A. koreana* essential oils appeared colourless to golden yellow to the unaided eye (Figure 1).

The major chemical profile of *A. koreana* oils is presented in Table 1. The oils were composed predominantly of monoterpenes (69.85%), followed by sesquiterpenes (11.05%). In particular, monoterpene hydrocarbons (51.72%) were the most abundant fraction in the total oils. The major chemical components of *A. koreana* oils were *D*-limonene (22.60%), bornyl acetate (15.02%), camphene (14.04%), α-pinene (11.96%) and β-eudesmol (11.05%), which together comprised 74.67% of the total oils.

### 2.2. Distillation Time Effects on the Oil Yield of A. koreana

**Figure 2:**
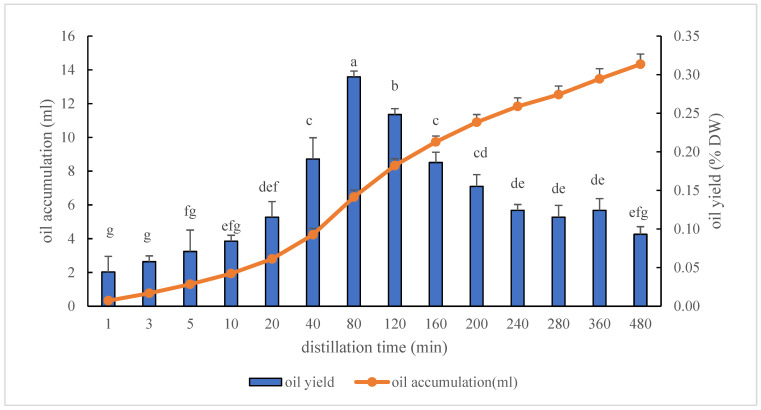
Fractional oil yield (%) and cumulative oil volume (mL) of *A. koreana* essential oils during 8 h of hydrodistillation. Values are expressed as means ± SD (*n* = 3). Values with different letters are significantly different at *p* < 0.05 as analysed by Tukey’s multiple range test of specialised fractional distillation for achieving the targeted yield. Fractional yield represents the percentage of oil obtained in each distillation interval relative to the dry weight, while cumulative volume indicates the total collected oil over time.

The effect of the DT on *A. koreana* oil yield and cumulative oil is presented in Figure 2. The oil yield (% DW) increased significantly with DT, showing a rapid rise during the early extraction stage (80 min). Within the first 40 min, the oil yield increased sharply, reaching 0.19 ± 0.03%, which was significantly higher than that obtained at shorter DT (1–20 min). The highest oil yield was observed at 80 min (0.30 ± 0.01%), after which a gradual decline was noted despite continued distillation. Specifically, oil yields decreased slightly and showed no significant differences between 200 and 480 min with a further reduction. When expressed as the oil yield per unit time, the extraction rate at 80 min (0.081 mL min^−1^) was approximately 33% higher than that at 160 min (0.061 mL min^−1^), suggesting that the efficiency of oil recovery declines as the distillation time increases. By contrast, the cumulative oil accumulation continuously increased throughout the distillation process (1–480 min). Oil accumulation increased slowly during the initial stage (1–20 min) but rose markedly between 40 and 160 min, corresponding to the period of the highest oil yield. After 160 min, the rate of oil accumulation gradually decreased, indicating a diminishing extraction rate; however, the total oil volume continued to increase until 480 min, reaching approximately 14.5 mL. Compared with the control, approximately 67.6% of the total potential essential oil yield was recovered after 8 h of distillation.

### 2.3. Effects of the Length of the Distillation Time on the Targeted Components of A. koreana Oils

As shown in Table 2, the targeted bioactive components (α-pinene, *D*-limonene, borneol, and bornyl acetate) in the *A. koreana* oils collected at different hydrodistillation times were identified.

The relative content of α-pinene decreased rapidly during the early stages of distillation, declining from 18.36 ± 3.62% at 1 min to 8.26 ± 0.75% at 20 min. Thereafter, its proportion gradually increased, reaching 15.35 ± 0.18% at 280 min, followed by a slight decrease toward the end of the distillation process. Similarly, *D*-limonene exhibited a continuous decreasing trend with prolonged DT, declining from 33.05 ± 1.16% at 1 min to 15.22 ± 0.81% at 480 min. In contrast, oxygenated monoterpenes, including borneol and bornyl acetate, showed distinct accumulation patterns. The borneol content increased rapidly during the early distillation period, reaching a maxima of 3.90 ± 0.51% and 3.84 ± 0.50% at 20 and 40 min, respectively, and subsequently declined with extended DT. Bornyl acetate exhibited a pronounced increase from 8.55 ± 4.32% at 1 min to peak levels between 20 and 40 min (23.19 ± 1.28–23.74 ± 0.82%), followed by a gradual decrease after 80 min. The total content of the targeted bioactive components remained relatively stable during the early and intermediate distillation stages (1–160 min), ranging from 60.18 ± 2.54% to 62.42 ± 3.62%. However, a marked decline was observed after 200 min, with the proportion decreasing to 39.20 ± 3.73% at 480 min.

### 2.4. The Distillation Condition of A. koreana Oils for Functional Cosmetic Applications

Based on the preliminary PCA, the 120 min oil clustered between the 80 and 160 min oils and did not exhibit a distinct chemical or fragrance profile. Therefore, it was excluded from the subsequent PCA to enhance the discrimination between representative oils with clearly differentiated extraction characteristics.

**Figure 3:**
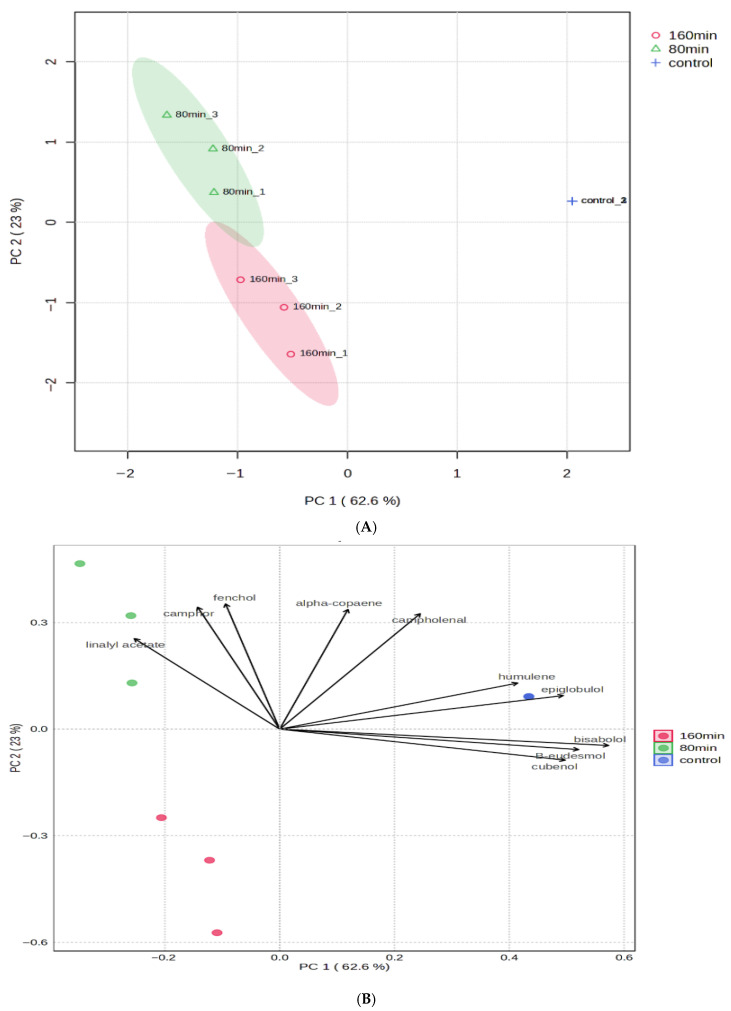
Principal component analysis (PCA) of three *A. koreana* oil samples. (**A**) The score plot of the PCA. (**B**) The biplot of PCA. Three essential oils sample: 80 min distillation, 160 min distillation, and the control.

From a sensory perspective, to further distinguish the fragrance differences between the essential oils obtained at 80 min, 160 min, and the control, a PCA was performed based on their chemical profiles. As shown in Figure 3, the accumulated contribution rate of the first two principal components (PC 1: 62.6%, PC2: 23%) was 85.6% of the total variance, indicating that the PCA model sufficiently represented the variability among the samples and allowed for clear discrimination.

**Figure 4:**
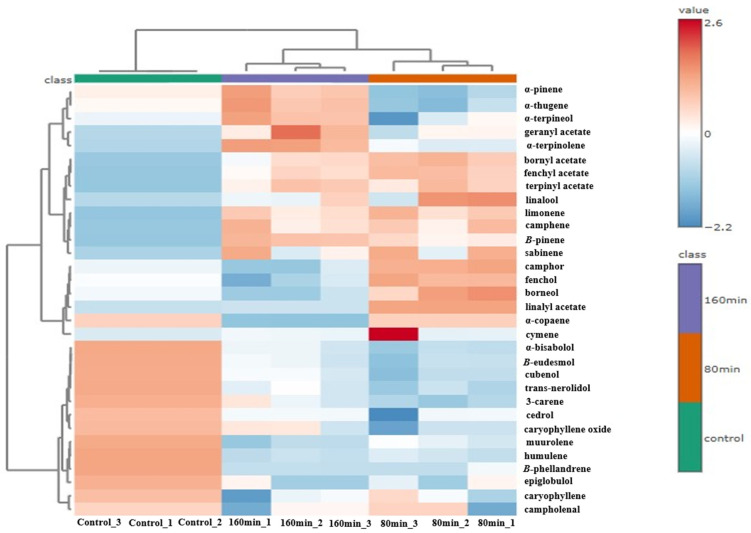
A hierarchical clustering heatmap of the major volatile compounds in the essential oils obtained at different distillation times (control vs. 80 min vs. 160 min). Data were log-transformed and auto-scaled before clustering. The Euclidean distance and Ward’s linkage method were used.

The essential oils extracted after 80 min distillation exhibited high scores on the positive side of PC2, where the loadings of characteristic volatile compounds included linalyl acetate, camphor and fenchol. In contrast, the essential oils from the 160 min distillation were characterised by negative scores on PC1 and were associated with compounds including bisabolol and β-eudesmol, showing a compositional trend closer to that of the control (Figure 4).

The control oils are exhibited high scores on the positive PC1 and PC2, where the loadings of characteristic volatile compounds included α-copaene, campholenal, humulene, and epiglobulol (Figure 4). Overall, the PCA revealed clear compositional differences among the three essential oils, while the oils obtained after 160 min of distillation showed greater similarity to the control compared to those obtained after 80 min.

## 3. Discussion

### 3.1. The Oil Yield and Chemical Profile of A. koreana Oils Extracted by Hydrodistillation

The essential oil of *A. koreana* used as the control in this study was extracted until no further oil could be obtained. The essential oil of *A. koreana* exhibits a prominent green note characterised by notable volume, intensity, and longevity. The initial impression is dominated by a leafy green note, gradually accompanied by terpenic and slightly fatty nuances, followed by a fresh citrus-like aroma [16]. The control oil yield was 2.82%, and *A. koreana* oils was dominated by monoterpenes (69.85%) and sesquiterpenes (11.05%) with monoterpenes as the major class (51.72%). The major chemical components of the *A. koreana* oil were *D*-limonene (22.60%), bornyl acetate (15.02%), camphene (14.04%), and *B*-eudesmol (11.05%), together accounting for 62.71% of the total oil (Table 1).

Essential oils have a very high variability in their composition, both in qualitative and quantitative terms owing to various factors, including, but not limited to, seasonal, geographical, and genetic differences [17]. Furthermore, a comparison of oil yields with those reported in previous studies is challenging, as differences in distillation methods can significantly influence both the yield and quality of the essential oils [18]. *A. koreana* is distributed in the alpine regions of the southern Korean peninsula, including Mt Dukyu, Mt Chiri, and Mt Halla [19]. As shown in Table 1, the chemical profile of the *A. koreana* oil was similar to that reported for the *A. koreana* oils originating from Mt. Halla in Jeju, 2007 [20]. Specifically, the needles of *A. koreana* were collected in June 2007 on Mt. Halla, and the samples were hydrodistilled for 6 h. The oil yield was 0.53 + 0.10% (*v*/*w*), and the major components of the oil were *D*-limonene (23.5%) and bornyl acetate (17.9%). On the other hand, the main components of the *A. koreana* oil from plants grown in Mt. Dukyu were borneol (27.9%), followed by α-pinene (23.2%). The needles were collected in November 2004 and the oil was hydrodistilled for 3 h with 0.9% (*v*/*w*) [7]. In particular, the variations in the oil yield and chemical profiles of *A. koreana* can be attributed to chemotypic diversity, which reflects intraspecific genetic variation and adaptive responses to local ecological conditions [21]. For the commercial production of *A. koreana* oil, comprehensive research based on an extensive collection of *A. koreana* from diverse locations in South Korea is important for selecting a premium cultivar with enhanced oil yield and bioactive components.

### 3.2. Effects of the Length of the Distillation Time on the Oil Yield of A. koreana Oils

Although distillation is a well-known and simple process, optimal conditions must be determined individually, as each plant material is unique and the conditions cannot be generalised [22]. As shown in Figure 2, the oil yield (0.04–0.30%) increased and reached a maximum at 80 min of distillation (0.30 ± 0.01%) when expressed as the yield obtained within each distillation interval. After 80 min, the amount of oil recovered in each subsequent interval gradually decreased. However, the cumulative oil yield continued to increase with extended distillation time, ultimately reaching 2.82% under exhaustive distillation conditions (control). At 80 min, approximately 10.6% of the total recoverable essential oil was obtained. Complete recovery of the remaining oil from the plant material would require a longer DT; however, prolonged distillation is associated with increased energy consumption, including but not limited to electricity, human labour, and maintenance. Therefore, further studies are required to develop strategies that reduce the DT while maintaining oil recovery efficiency. Under moderate pressure, the DT can be significantly shortened, leading to lower steam consumption and, consequently, reduced energy use. For example, aromatic plants such as sandalwood and cloves, as well as the rhizomes of vetiver, ginger, and iris, require more than 24 h under atmospheric distillation, whereas pressure steam distillation at 0.5 MPa reduces the extraction time to less than 3 h [23].

### 3.3. Effect of Distillation Time on the Selected Components of A. koreana Oils

Due to the complexity of this study and the large number of components present in the essential oils, it was necessary that we focused on the major and most economically important constituents present in *A. koreana* essential oils. Owing to the biological activities and unique fragrance, *A. koreana* essential oils have potentials as a safe and an effective skin ingredient for whitening and anti-wrinkle. Specifically, the key bioactive components in *A. koreana* oils are α-pinene, *D*-limonene, borneol, and bornyl acetate [8,24]. The aforementioned targeted bioactive components (α-pinene, *D*-limonene, borneol, and bornyl acetate) in *A. koreana* oils varied significantly depending on the hydrodistillation time. Chhibber et al. [25] reported that the order of component release during essential oil hydrodistillation is primarily dictated by compound polarity, as a result of simultaneous water–compound interactions throughout the distillation process. Furthermore, this polarity makes the compounds soluble in water, and this solubility is a function of the physical properties of the system such as pressure, temperature and chemical potential [26].

Many previous studies have shown that DT influences essential oil composition. Consistent with these reports, monoterpenes were predominantly eluted at the early distillation stages, whereas prolonged distillation resulted in oils enriched in sesquiterpenes [27]. The elution behaviour of individual compounds varies with plant species and distillation conditions [28,29]. In the case of garden sage (*Salvia officinalis* L.) [30], the monoterpene hydrocarbons (α-pinene, camphene, β-pinene, myrcene, and limonene) were eluted early in the steam distillation process, which resulted in their high concentrations in the oil at 5 to 10 min of DT and relatively low concentrations in the oil obtained at 160 min. In particular, monoterpene contents showed fluctuations during the early stages of distillation. This behaviour likely reflects the high volatility and chemical instability of monoterpenes, which are associated with the presence of C=C double bonds [31]. Interestingly, the relative proportion of α-pinene increased after 20 min, reaching 15.35% at 280 min (Table 2). This apparent increase does not necessarily indicate an increase in its absolute concentration, but rather reflects changes in the relative composition. One possible explanation is related to the plant matrix characteristics. Essential oil components stored within resin ducts or cellular compartments can be gradually released during prolonged distillation due to diffusion processes and the structural disruption of plant tissues [32]. Monoterpenes such as α-pinene are known to be associated with the volatile fraction of oleoresin stored in the resin ducts [33]. In *A. koreana*, these resin-containing structures are present not only in the stems and twigs but also in the needles, although in smaller quantities, which may contribute to the delayed release of α-pinene during extended hydrodistillation. In addition, the observed trend may be influenced by compositional redistribution arising from the relative normalisation of GC–MS data, where the depletion of other components leads to an apparent increase in the relative proportion of α-pinene. Therefore, the increase in α-pinene at longer distillation times is likely due to the combined effect of relative compositional redistribution and the gradual release from the plant matrix, rather than a true increase in its absolute concentration.

The concentration of sesquiterpenes (*B*-caryophyllene, α-humulene, and viridifloral) increased with the increasing duration of DT and reached its respective maximum concentration in the oil at 160 min. Furthermore, in juniper essential oils (*Juniperus scopulorum*), eudesmol, an oxygenated sesquiterpenes, reached a maximum (1.72%) at 480 min DT [34]. The combined content of the targeted bioactive components remained relatively stable during the early and intermediate distillation stages (1–160 min; 60.18–62.42%), but decreased markedly after 200 min, reaching 39.20% at 480 min. These results indicate that a distillation time of at least 160 min is required to maximise the accumulation of bioactive constituents associated with the whitening effects of *A. koreana* oils. Furthermore, the compositional shift suggested that prolonged distillation favours the extraction of less volatile, higher molecular weight compounds rather than degradation or artefact formation. Hence, the decrease in the targeted bioactive monoterpenes does not represent a loss of total functionality, but rather a transition toward a sesquiterpene-enriched oil with potentially different bioactive properties (Figure 3). Therefore, the precise manipulation of distillation time represents a cost-effective strategy for producers to obtain essential oils with tailored chemical profiles. Nevertheless, further studies are required to directly evaluate the whitening and anti-wrinkle properties of *A. koreana* oils obtained at different DTs, as essential oils are complex mixtures and potential synergistic interactions among components may play a critical role in their biological efficacy [35].

### 3.4. The Distillation Condition of A. koreana Oils as a Functional Cosmetic Ingredient

Understanding the effects of DT on essential oil yield and composition might allow producers of these economically important commercial oils to increase production and engineer the composition of the oils while decreasing the energy required for distillation [15]. To contribute to the potential commercialisation of essential oils, the production process must be aligned with conditions that ensure a good process and high quality. One of the main challenges in scaling up essential oil production is the extraction process, due to its lengthy time and high energy demands [36]. Therefore, manipulating the DT by jointly considering oil yield, targeted bioactive components, and fragrance characteristics is essential for the development of *A. koreana* essential oils as bioactive cosmetic ingredients. To achieve efficient oil recovery, *A. koreana* oil can be distilled for 80 min, at which the oil fraction reaches its maximum (0.30 ± 0.01%) (Figure 2). Assuming this, the cumulative oil collected over 0–80 min was 6.47 mL (including an additional 0.42 mL), obtained from 751 g of dried sample, corresponding to a yield of 0.86% with returns beyond 80 min. Yet, the distillation condition can differ to enhance the targeted bioactive components in *A. koreana* oil. DT of at least 160 min is required to maximise the accumulation of bioactive constituents associated with the whitening effects of *A. koreana* oils (Table 2). The chemical composition of lemongrass essential oils varied with DT, indicating its potential to be tailored to specific applications. The components of neral and geranial were maximised between 10 and 40 min, coinciding with the DT for optimal oil yield, whereas caryophyllene oxide and trans-caryophyllene increased with prolonged DT. A distillation time of 20 min optimised the oil yield and the concentrations of neral and geranial, while maintaining low levels of caryophyllene oxide (<2%) and trans-caryophyllene (<1%). Furthermore, in peppermint oil, undesirable components such as menthofuran can be minimised by controlling the DT. Therefore, DT can be used as an effective tool to obtain essential oils enriched in specific target bioactive components.

The PCA and heatmap analyses revealed distinct differences in the chemical composition of the essential oils distilled at 80 min, 160 min, and the control, which were closely associated with variations in their fragrance characteristics (Figure 3 and Figure 4). Fragrances play a crucial role in enhancing the attractiveness of cosmetic products, as pleasant scents influence user comfort, perceived product efficacy, and the overall evaluation of the cosmetics [37]. Monoterpenes and ester compounds, which are known to contribute to fresh and highly volatile top-note aromas, are typically extracted at the early stages of distillation, whereas sesquiterpenes and oxygenated sesquiterpenes impart woody, balsamic, and long-lasting aroma characteristics due to their lower volatility [38,39]. From a sensory perspective, the modulation of the DT enables the tailoring of *A. koreana* oil aroma profiles, allowing for the selective production of oils with fresh top-note dominance (80 min), owing to abundant monoterpenes or more balanced and persistent aroma characteristics owing to higher sesquiterpenes (160 min). The control samples were dominated by sesquiterpenes and oxygenated sesquiterpenes, which are known to impart woody, balsamic, and long-lasting aroma characteristics [37,40]. Nevertheless, although DT modulation enables precise control over aroma characteristics, its practical application in industrial settings must consider the associated energy requirements and economic constraints. A better agricultural output depends on the right kind of energy being available in adequate amounts and utilised efficiently [41,42]. Extending the DT to maximise the targeted bioactive components in the oils would be a difficult decision for manufacturers due to the additional production costs. However, the application of essential oils as functional cosmetic ingredients offers substantially greater added value compared with their use as raw essential oils alone. Therefore, further research is required to perform comprehensive economic evaluations of *A. koreana* oils to support the development of a sustainable and profitable industry [43,44]. Additionally, the results of this laboratory-scale study cannot be directly extrapolated to industrial essential oil production, as industrial distillation systems operate under substantially different process conditions and equipment scales, which may influence both extraction efficiency and chemical composition [45].

## 4. Materials and Methods

### 4.1. Chemical Reagents

C7–C40 saturated alkane standard mix (Lot #LRAC3115) was sourced from Sigma-Aldrich, St Louis, MO, USA. Anhydrous sodium sulfate (98.5%, Samchun, Seoul, Republic of Korea) was used.

### 4.2. Plant Material and Essential Oil Hydrodistillation

Wild *Abies koreana* samples were collected from a plantation in Haessal Village, Inje, Gangwon Province (38.01287188° N, 128.1941453° E), Republic of Korea (October 2025). The characteristics of the sampling site were obtained via weather extrapolation. The database and tool constitute a comprehensive archive of climate data recorded by the Korea Meteorological Administration National Climate Data Centre. Based on the climate data for October 2025, the mean minimum, mean, and maximum temperatures were 10.6 °C, 14.0 °C, and 18.6 °C, respectively, with a total precipitation of 123.8 mm [46].

*A. koreana* trees (approximately 10 years old; plant height ≈ 3 m) were used in this study. The trial was conducted using a randomised complete block design (RCBD) with three blocks. Approximately 2 kg of *A. koreana* samples were collected from individual trees within each block. Plant materials were obtained through pruning and thinning processes as a non-destructive method. Only healthy needles, free from drying, discoloration, and pest or disease damage, were carefully selected and collected for the experiment. The sampled plant was cut and labelled in the field before being stored on ice in a thermally resistant container for transport to the laboratory. The essential oil was extracted from the needles together with small twigs (diameter < 1 cm) of *A. koreana*. The needles were separated from larger branches prior to extraction, and only the needles and fine twigs were included as the extraction material.

The samples were then weighed, and sub-samples were dried at 105 °C for 24 h to determine oven-dry weight (ODW). The remaining material was packed in plastic bags and stored at −18 °C until extraction by hydrodistillation.

The essential oils were extracted from the leaves via hydrodistillation using a Clevenger apparatus. Essential oil extraction was performed at the National Institute of Forest Science (Seoul, Republic of Korea). Specifically, each sample (1.6 kg) was mixed with distilled water (DW) in a ratio of 1:7 (kg:L). Samples soaked in the DW were heated using a heating mantle (Model: MS-DM608, Serial number: 201602, Misung Scientific Co., Ltd., Yangju, Republic of Korea) to approximately 102 °C, which corresponds to the boiling temperature of the water-plant mixture under the experimental conditions. Distillation was continued until no further oil was obtained (20 h), and the resulting sample was used as the control. The current experimental design was modified according to Zheljazkov et al. [47]. To investigate the DT effect on essential oils, samples were taken at 1, 3, 5, 10, 20, 40, 80, 120, 160, 200, 240, 280, 360, and 480 min. Specifically, essential oil fractions were collected at 14 sequential distillation time (DT) intervals (0–1 min, 1–3 min, 3–5 min, 5–10 min, 10–20 min, 20–40 min, 40–80 min, 80–120 min, 120–160 min, 160–200 min, 200–240 min, 240–280 min, 280–360 min, and 360–480 min), along with a control. The internal volume of the collection arm was minimal, and no visible oil residue remained after collection. The oils were obtained and analysed separately at the given 14 different DTs. Each fraction was taken from the same Clevenger apparatus. The yield of the essential oils was calculated using the following equation.

Essential oil yield (%) = [essential oils distilled (mL)/sample weight (ODWg)] × 100%.

The essential oils were dehydrated using an anhydrous sodium sulfate (98.5%, Samchun, Seoul, Republic of Korea) and stored in refrigerator at 4 °C until use.

### 4.3. Analysis of Gas Chromatography-Mass Spectrometry (GC-MS)

The volatile constituents of the essential oils were analysed using GC–MS (7890B GC system coupled with a 5977A MSD, Agilent Technologies, Inc., Santa Clara, CA, USA) equipped with a VF-5MS capillary column (60 m × 0.25 mm, 0.25 µm; Agilent Technologies). The temperature of the GC injector was set to 280 °C, and the flow rate of the helium carrier gas was 2.0 mL/min. The initial oven temperature was 50 °C (5 min), followed by a temperature increase to 65 °C (30 min) at 10 °C/min. Thereafter, the temperature was raised to 210 °C (10 min) at 5 °C/min and, finally, to 305 °C (5 min) at 20 °C/min. The MS was conducted in the electron ionisation mode (70 eV). The ion source and interface temperatures were set to 270 °C and 250 °C, respectively, and a mass range of 35–550 amu was recorded in the full-scan mode. The Kovats retention indices (KI) of the individual compounds were evaluated by comparing their relative retention times with those of an n-alkanes mixture (C8–C30, Sigma-Aldrich, St. Louis, MO, USA) in a VF-5MS column. The volatile constituents were identified by comparing their calculated KIs with the reported values (e.g., the NIST Chemistry WebBook). All GC–MS analyses were conducted at the National Instrumentation Centre for Environmental Management (NICEM), Seoul National University, Seoul, Republic of Korea.

### 4.4. Multivariate Statistical Analysis

The multivariate statistical analyses were performed according to references [48,49] with modifications. Multivariate statistical analysis was conducted to evaluate the chemical composition of *A. koreana* oils. Based on the preliminary principal component analysis (PCA), the 120 min oil clustered between the 80 and 160 min oils and did not exhibit a distinct chemical or fragrance profile; therefore, it was excluded from subsequent PCA to improve the discrimination among representative samples. PCA was then performed on the chemical profiles of oils obtained at 80 min, 160 min, and the control sample from exhaustive distillation (20 h), to further differentiate their fragrance characteristics.

GC–MS data, expressed as relative peak area percentages, were organised into a data matrix and normalised to total ion abundance, followed by autoscaling (mean-cantered and unit variance). PCA was performed as an unsupervised method to visualise compositional differences among the essential oil samples extracted at different distillation times. PCA score plots were used to assess sample clustering, while PCA biplots illustrated the contribution of individual volatile compounds to sample separation. The variance explained by each principal component was calculated. Hierarchical clustering analysis was carried out using auto-scaled data, with Euclidean distance and Ward’s linkage method [50,51]. Heatmaps were generated to visualise relative compound abundance across samples, with rows representing volatile compounds and columns representing biological replicates. Analyses were performed using MetaboAnalyst software (version 6.0; University of Alberta, Edmonton, AB, Canada).

### 4.5. Statistical Analysis

Data is presented as means ± SD (*n* = 3), except for the control oil (*n* = 1). One limitation of this study is that the control sample obtained by exhaustive hydrodistillation (20 h) was analysed as a single replicate (*n* = 1), due to the limited availability of plant material. Consequently, the control was not included in the statistical analyses and was used only as a qualitative reference. Although it was included in the PCA to provide a comparative anchor for fragrance and compositional differences, the results involving the control should be interpreted with caution.

The differences in oil yield and the chemical profile of the oils were analysed using a one-way ANOVA followed by post hoc Tukey’s multiple range tests (Version 9.4; SAS Institute Inc., Cary, NC, USA). Furthermore, multivariate statistical analysis was performed using MetaboAnalyst software (version 6.0; University of Alberta, Edmonton, AB, Canada).

## 5. Conclusions

For commercial production, understanding and optimising the DT is crucial for maximising oil yield and producing premium-quality oils. This study is the first to evaluate the effect of DT on *A. koreana* essential oils to increase oil yield and enrich targeted bioactive components for commercial applications.

The control oil, obtained by hydrodistillation for 20 h, yielded 2.82% and was dominated by monoterpenes (69.85%) and sesquiterpenes (11.05%), with *D*-limonene, bornyl acetate, camphene, and *B*-eudesmol as the major constituents. The key bioactive components (α-pinene, *D*-limonene, borneol, and bornyl acetate), known for their whitening and anti-wrinkle activities, varied significantly with the DTs. Accordingly, the distillation conditions were optimised by jointly considering the oil yield and targeted component abundance. The oil yield increased rapidly during the early stages of distillation and reached a point of diminishing returns around 80 min (0.30 ± 0.01%), beyond which the rate of oil recovery became substantially lower. In contrast, oils enriched in the targeted bioactive components relevant to whitening and anti-wrinkle activities were obtained at longer distillation times (80–160 min). DT also significantly influenced fragrance characteristics: oils distilled for 80 min were rich in monoterpenes (linalyl acetate, camphor, and fenchol) and exhibited fresh, top-note-dominant aromas, whereas longer DTs (≈160 min) increased sesquiterpene content (bisabolol and β-eudesmol), resulting in more persistent fragrance profiles.

Therefore, the distillation time of *A. koreana* oil exerted a significant influence on both oil yield and bioactive composition, with shorter distillation (80 min) favouring oil yield maximisation, whereas longer distillation times (80–160 min) promoted the enrichment of bioactive components relevant to cosmetic applications. Further research is required to evaluate the economic implications of distillation time, including labour, energy consumption, and production costs. Furthermore, future comparative studies should investigate improved methodologies for essential oil extraction, including advanced techniques such as ultrasound-assisted extraction (UAE), microwave-assisted hydrodistillation (MAHD), pressurised solvent extraction, ultrasound-assisted extraction, and supercritical CO_2_ extraction, in order to evaluate their efficiency, selectivity, and extraction time relative to conventional hydrodistillation. Ultimately, precise control of DT enables the production of tailored, high-quality oils with enhanced bioactive potential for cosmetic and industrial applications.

## Figures and Tables

**Table 1:** The chemical profile of *A. koreana* oils.

RT (min) *	KI *	KI Ref *	Chemical Constituents	% in Oils
12.85	908	916	tricyclene	0.01
13.76	925	925	α-thugene	1.51
14.30	935	931	α-pinene	11.96
15.22	952	940	camphene	14.04
16.73	981	963	sabinene	1.60
17.21	990	968	β-pinene	2.07
18.46	1012	1011	3-carene	0.46
19.44	1027	1029	cymene	0.04
19.75	1032	1031	*D*-limonene	22.60
20.66	1047	1037	β-phellandrene	0.08
23.24	1088	1088	α-terpinolene	0.39
24.08	1102	1098	linalool	0.30
25.56	1124	1119	fenchol	0.07
27.49	1153	1143	camphor	0.12
29.09	1178	1168	borneol	2.44
30.54	1200	1189	α-terpineol	0.25
31.94	1220	1219	fenchyl acetate	1.06
33.89	1249	1130	campholenal	0.10
35.18	1268	1257	linalyl acetate	0.01
36.48	1287	1285	bornyl acetate	15.02
41.24	1355	1350	terpinyl acetate	0.55
42.76	1378	1373	α-copaene	0.02
43.18	1384	1386	geranyl acetate	0.13
45.55	1415	1418	caryophyllene	2.95
47.13	1465	1451	humulene	0.60
48.29	1492	1505	muurolene	4.71
50.55	1558	1560	epiglobulol	0.07
50.81	1566	1560	trans-nerolidol	0.86
51.74	1595	1581	caryophyllene oxide	0.06
52.6	1625	1601	cedrol	0.03
52.95	1638	1643	cubenol	0.62
54.18	1684	1580	β-eudesmol	11.05
54.56	1698	1701	α-bisabolol	1.10
Total identified components (%)			96.85	
Unknown components (%)			3.15	

RT *: Retention time (min), KI *: Kovats index; KI ref *: KI reference from the NIST Chemistry WebBook.

**Table 2:** The effect of distillation time on the targeted bioactive components in the *A. koreana* oils during eight hours of distillation.

Time (min)	The Targeted Components (% in Oils)				
	α-Pinene	*D*-Limonene	Borneol	Bornyl Acetate	(+)
1	18.36 +3.62 ^a^	33.05 + 1.16 ^a^	1.07 + 0.59 ^f^	8.55 + 4.32 ^g^	61.04 + 9.70 ^a^
3	13.92 + 0.41 ^bcd^	32.56 + 1.00 ^a^	1.79 + 0.34 ^def^	13.24 + 1.03 ^fg^	61.51 + 2.78 ^a^
5	11.79 + 0.39 ^cde^	29.14 + 1.36 ^b^	2.57 + 0.12 ^bcd^	16.79 + 0.62 ^cde^	60.29 + 2.49 ^a^
10	10.37 + 0.71 ^ef^	26.63 + 0.52 ^c^	3.19 + 0.20 ^ab^	20.27 + 1.07 ^abc^	60.46 + 2.50 ^a^
20	8.26 + 0.75 ^f^	27.08 + 1.08 ^bc^	3.90 +0.51 ^a^	23.19 + 1.28 ^a^	62.42 + 3.62 ^a^
40	8.62 + 0.35 ^f^	25.75 + 0.75 ^cd^	3.84 + 0.50 ^a^	23.74 + 0.82 ^a^	61.95 + 2.41 ^a^
80	10.04 + 0.30 ^ef^	26.24 + 0.70 ^c^	3.06 + 0.26 ^abc^	22.41 + 0.84 ^ab^	61.74 + 2.10 ^a^
120	11.53 + 0.26 ^def^	26.21 + 0.40 ^cd^	2.43 + 0.13 ^bcde^	21.07 + 1.23 ^abc^	61.24 + 2.02 ^a^
160	12.83 +0.52 ^bcde^	25.51 + 0.46 ^cd^	2.07 + 0.12 ^cdef^	19.78 + 1.43 ^abcd^	60.18 + 2.54 ^a^
200	14.38 + 1.19 ^bcd^	24.81 + 0.51 ^cd^	1.81 + 0.26 ^def^	17.53 + 2.58 ^bcde^	58.53 + 4.54 ^ab^
240	15.09 + 0.74 ^abc^	23.87 + 0.56 ^de^	1.65 + 0.20 ^def^	15.70 + 1.78 ^def^	56.32 + 3.28 ^ab^
280	15.35 + 0.18 ^ab^	22.27 + 0.38 ^e^	1.58 + 0.22 ^def^	14.58 + 1.32 ^def^	53.78 + 2.09 ^ab^
360	14.28 + 0.40 ^bcd^	19.23 + 0.41 ^f^	1.52 + 0.30 ^ef^	13.46 + 1.64 ^efg^	48.50 + 2.75 ^bc^
480	12.05 +0.45 ^bcde^	15.22 + 0.81 ^g^	1.29 + 0.44 ^f^	10.63 + 2.04 ^fg^	39.20 + 3.73 ^c^

Values are expressed as means ± SD (*n* = 3). Values with different superscript letters are significantly different at *p* < 0.05, as analysed by Tukey’s multiple range test for specialised fractional distillation to achieve the targeted yield.

## Data Availability

The original contributions presented in this study are included in the article. Further inquiries can be directed to the corresponding author.

## Abbreviations

The following abbreviation is used in this manuscript:

DT	Distillation Time
